# Differential Expression in Testis and Liver Transcriptomes from Four Species of *Peromyscus* (Rodentia: Cricetidae)

**DOI:** 10.1093/gbe/evz280

**Published:** 2020-01-07

**Authors:** Laramie L Lindsey, Roy N Platt, Caleb D Phillips, David A Ray, Robert D Bradley

**Affiliations:** 1 Department of Biological Sciences, Texas Tech University; 2 Genetics Department, Texas Biomedical Research Institute, San Antonio, Texas; 3 Natural Science Research Laboratory, Museum of Texas Tech University

**Keywords:** comparative genomics, differential expression, lineage diversification, *Peromyscus*, transcriptome

## Abstract

The genus *Peromyscus* represents a rapidly diverged clade of Cricetid rodents that contains multiple cryptic species and has a propensity for morphologic conservation across its members. The unresolved relationships in previously proposed phylogenies reflect a suspected rapid adaptive radiation. To identify functional groups of genes that may be important in reproductive isolation in a reoccurring fashion across the *Peromyscus* phylogeny, liver and testis transcriptomes from four species (*P*. *attwateri*, *P*. *boylii*, *P*. *leucopus*, and *P*. *maniculatus*) were generated and differential expression (DE) tests were conducted. Taxa were selected to represent members diverged from a common ancestor: *P*. *attwateri* + *P*. *boylii* (clade A), and *P*. *leucopus* + *P*. *maniculatus* (clade B). Comparison of clades (A vs. B) suggested that 252 transcripts had significant DE in the liver data set, whereas significant DE was identified for 657 transcripts in the testis data set. Further, 45 genes had DE isoforms in the 657 testis transcripts and most of these functioned in major reproductive roles such as acrosome assembly, spermatogenesis, and cell cycle processes (meiosis). DE transcripts in the liver mapped to more broad gene ontology terms (metabolic processes, catabolic processes, response to chemical, and regulatory processes), and DE transcripts in the testis mapped to gene ontology terms associated with reproductive processes, such as meiosis, sperm motility, acrosome assembly, and sperm–egg fusion. These results suggest that a suite of genes that conduct similar functions in the testes may be responsible for the adaptive radiation events and potential reoccurring speciation of *Peromyscus* in terms of reproduction through varying expression levels.

## Introduction

Nascent species resulting from adaptive radiations often display substantive morphological differences (Losos and Miles 2002); however, structural and functional genomic changes also may facilitate adaptive divergence without overt morphological differences (Kirkpatrick and Barton 2006; Rowe et al. 2011). The genus *Peromyscus* may exemplify the second scenario with ≥70 species hypothesized to have arisen during the last 5–6 Myr (Bradley et al. 2007; Platt et al. 2015). Differential expression (DE) studies have been conducted on *Peromyscus* to determine how variations in gene expression contribute to challenging environments, behavioral characteristics of *Peromyscus*, and mating systems (all reviewed by Munshi-South and Richardson [2017]); however, to date, no studies have examined the effects of gene expression patterns on potential mode(s) of speciation of the group.

Determining causes of speciation may be achieved by focusing on evolutionary forces, ecological circumstances, and genetic mechanisms (Marie Curie SPECIATION Network 2012). Further, gene expression plays a significant role in evolutionary processes and speciation (Galili et al. 1988; Pfennig et al. 2010); however, many obstacles exist to fully understand this phenomenon including technological restrictions (Wolf et al. 2010). With advances in technology, some researchers have been able to quantitatively examine DE in relation to speciation. For example, Brill et al. (2016) examined gene expression in adult testes of two allopatric species of Hawaiian *Drosophila* and discovered differentially expressed genes involved in sperm production between the two species that potentially led to the speciation of the Hawaiian *Drosophila* species.

Despite low levels of genetic divergence in *Peromyscus*, differentiation must have occurred to generate the reproductively isolated lineages. This genetic variation may accumulate by either differences in sequences at the base pair level or differences in the expression of genes or their isoforms. It is crucial to determine whether the processes are a result of continual selective forces on the genome at the early stages of radiation or if mutations/DE occurred in a random set of genes for each member of the 13 clades in *Peromyscus* (see Bradley et al. 2007; Platt et al. 2015). Were genes with functional similarities under selection pressures in a reoccurring fashion for each cladogenic event, or did changes occur in a stochastic manner? We hypothesize that genes associated with similar functions of reproduction (i.e., spermatogenesis and sperm motility) will be differentially expressed at the ancestral nodes for major *Peromyscus* clades and may reveal potential mode(s) of speciation for the entire group. Further, we hypothesize that DE would occur in “nonreproductive” genes stochastically.

To address these hypotheses, we examined transcriptome libraries from liver and testes. Presumably, liver is responsible for a broad assortment of biological processes (BPs), including metabolic and cellular activities (Kampf et al. 2014; Uhlén et al. 2015), whereas testes are involved in reproductive processes (Eddy 2002); reproductive genes evolve rapidly and are thought to influence speciation (Swanson and Vacquier 2002). We assume that genes expressed in the liver will represent a broader set of BPs (cell, multicellular, organismal, physiological, structural modifications, etc.) as measured by gene ontology terms (GO terms: Ashburner et al. 2000; Gene Ontology Consortium 2019) than testes (cell cycle, meiotic, reproduction, etc.). We also assume that genes expressed in the testes are more likely to play a role in processes associated with speciation events than those from the liver, and therefore predict that comparisons of species of *Peromyscus* from different phylogenetic clades would possess a greater number of DE genes in the testes data set (with less general GO terms) than in the liver data set (with more broad GO terms). However, given that we can only hypothesize about general patterns in the testis and liver, results may be indicative of either genetic drift or selection. Further, it is difficult to determine how genes expressed in the liver would potentially contribute to the adaptive radiation processes.

**Fig. 1.**
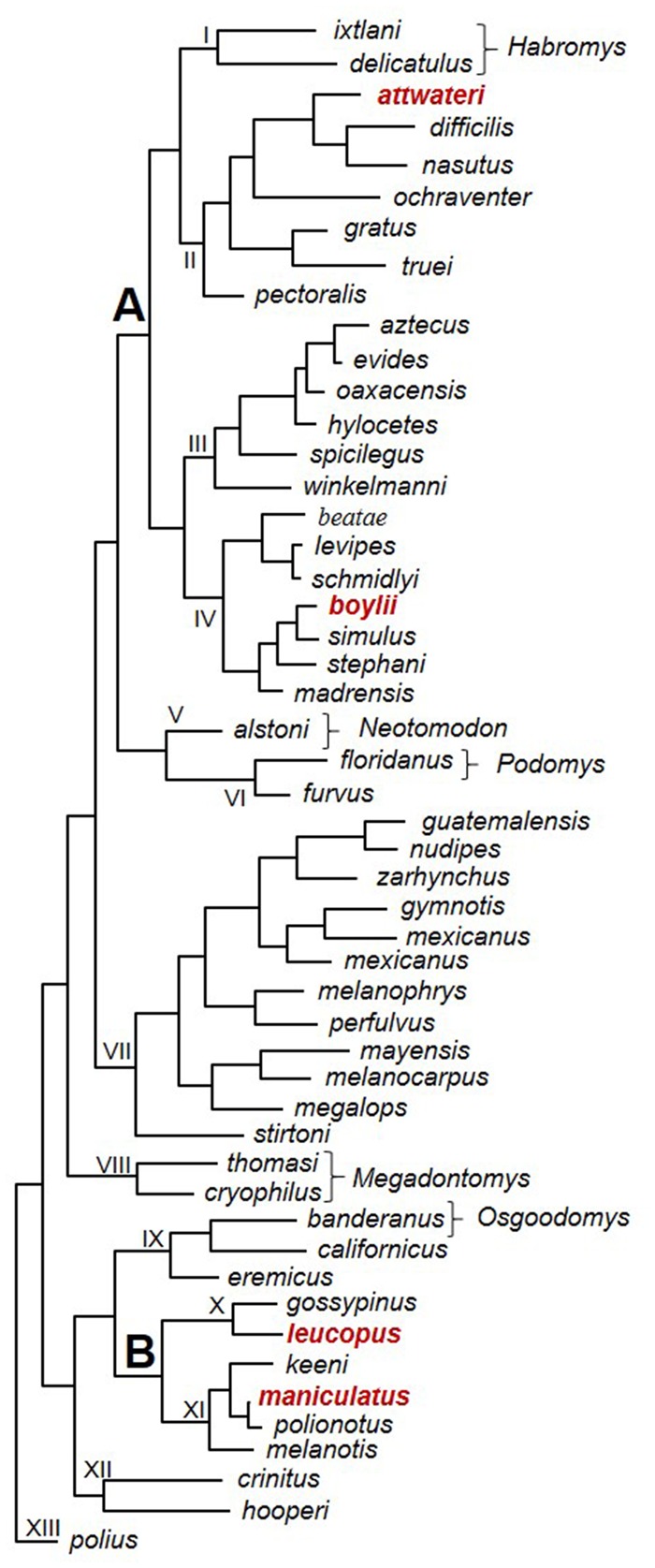
—Proposed phylogenetic relationships among *Peromyscus* derived from Bradley et al. (2007) and Platt et al. (2015) (for statistical support of nodes, review the articles). Common ancestor A contains *P*. *attwateri* and *P*. *boylii* as replicates, and common ancestor B contains *P*. *leucopus* and *P*. *maniculatus* as replicates. The four species sampled in this study are bolded and in red in the tree. Roman numerals have been added to indicate species groups as envisioned by Carleton (1989) and Bradley et al. (2007): I, subgenus *Habromys*; II, *truei* species group; III, *aztecus* species group; IV, *boylii* species group; V, subgenus *Neotomodon*; VI, subgenus *Podomys* and *furvus* species group; VII, *mexicanus*, *melanophrys*, and *megalops* species groups; VIII, subgenus *Megadontomys*; IX, subgenus *Osgoodomys*, *californicus*, and *eremicus* species groups; X, *leucopus* species group; XI, *maniculatus* species group; XII, *crinitus* and *hooperi* species groups; XIII, *polius* species group.

The goals of this project were 1) to examine genes that are differentially expressed between common ancestors of *Peromyscus* and determine if similar reproductive genes are expressed in a reoccurring fashion (based on testis transcriptomes) and 2) to determine if nonreproductive genes are differentially expressed randomly in terms of functionality as measured by GO terms (based on liver transcriptomes). To address these goals, liver and testis transcriptomes were obtained and assembled using a genome-guided approach (Grabherr et al. 2011) from clades with varying levels of phylogenetic relationships. Because DE may be related to branch lengths/node depth in the *Peromyscus* tree, four species (*P*. *attwateri*, *P*. *boylii*, *P*. *leucopus*, and *P*. *maniculatus*) were chosen based on their position in the *Peromyscus* phylogenetic tree (fig. 1).

## Materials and Methods

### Experimental Design

Taxonomic groups were selected from divergent portions of the *Peromyscus* phylogeny (fig. 1; see Bradley et al. 2007; Platt et al. 2015) to maximize the probability of selecting genes that were functionally important in speciation. For example, if sister species were selected, then we would predict that we would enrich for genes involved in speciation for those two taxa; whereas, samples selected from the membership of divergent clades would enhance the probability of identifying genes involved in early stages of radiation. Two taxa (*P*. *attwateri* and *P*. *boylii*) were selected from clade II and clade IV, respectively (fig. 1). *Peromyscusattwateri* and *P. boylii* share a common ancestor ∼3.5 Ma (Platt et al. 2015) and act as replicates for members diverged from common ancestor A (fig. 1). Furthermore, two taxa (*P*. *leucopus* and *P*. *maniculatus*) were selected from clade X and clade XI, respectively (fig. 1). The second pair has a more recent divergence, ∼2.5 Ma, and individually act as replicates for members diverged from common ancestor B (fig. 1). Liver tissues were chosen as controls because functions of the liver include metabolic control and detoxification which are more commonly associated with diet and overall “house-keeping” of the body.

### RNA Extraction, Library Prep, and Sequencing

Liver and testis tissues were obtained (and frozen immediately in liquid nitrogen) from scrotal males of four species (*P. attwateri*, *P. boylii*, *P. leucopus*, and *P. maniculatus*) during the summer of 2012, 2013, or 2014 and archived at the Natural Science Research Laboratory, Museum of Texas Tech University (table 1). Tissues were separately homogenized and RNA extracted using the TRIzol reagent (ThermoFisher) following the manufacturer’s recommended protocol. RNA quality was quantified using a Bioanalyzer System (Agilent Genomics) with a minimum of 7.5 for the RNA Integrity Number score. Sequencing libraries were generated from 500 ng of RNA from each sample using the NEB NEXT library prep kit (New England Biolabs, Beverly, MA). Indexed libraries were pooled in equimolar concentrations and paired-end, 100-bp reads were sequenced from the cDNA libraries using an Illumina HiSeq 2000. Illumina paired-end reads were clipped, trimmed, and orphans were sorted using Trimmomatic v0.27 (Bolger et al. 2014). To count the number of pairs and improper or orphaned read alignments, reads were aligned using the program, Bowtie2 v2.3.4 (Langmead and Salzberg 2012). In total, eight transcriptomes were generated, two for each individual, representing the liver and testis samples.

**Table 1:** Species and Tissue Types Used for De Novo and Genome-Guided Transcriptome Assemblies

Species	TTU-M/TK	Tissue Type	Total Reads	Genes	Isoforms	Alignment Rate (%)
*Peromyscus attwateri*	120500/181002	Liver	15,487,057	39,884	49,270	89.15
*Peromyscus attwateri*	120500/181002	Testis	17,997,884	52,863	85,226	83.25
*Peromyscus boylii*	118899/179930	Liver	14,608,016	43,218	67,236	86.33
*Peromyscus boylii*	118899/179930	Testis	19,824,124	68,983	116,403	83.76
*Peromyscus leucopus*	120519/181047	Liver	17,487,125	47,391	62,808	88.36
*Peromyscus leucopus*	120519/181047	Testis	17,095,228	67,170	98,305	82.57
*Peromyscus maniculatus*	140681/186091	Liver	23,803,797	43,013	54,772	89.93
*Peromyscus maniculatus*	140681/186091	Testis	28,187,132	109,623	157,143	83.18

Note.—The *Peromyscus maniculatus bairdii* genome was used as a reference in the genome-guided assembly. TTU-M and TK represent Texas Tech University Museum catalog number and Tissue Karyotype number, respectively. The genes, isoforms, and alignment rates (as predicted by RSEM results [Li and Dewey 2011]) are listed in the last three columns.

### Transcriptome Assembly and Annotation

A genome-guided assembly was implemented to annotate each transcriptome using the program, Trinity v r20150110beta (Grabherr et al. 2011) and the *Peromyscus maniculatus bairdii* genome assembly (GCA_003704035.1 HU_Pman_2.1). According to the Ensembl entry for the *Peromyscus maniculatus bairdii* genome, genes in the current draft of the genome assembly were aligned and predicted from homology searches with *Mus musculus*, as well as humans and other mammals (Aken et al. 2017). The *M. musculus* database was used for further analyses, including assigning Kyoto Encyclopedia of Genes and Genomes (KEGG) and GO terms, because the *M. musculus* database is more readily available in comparison to *P. maniculatus*. Further, a de novo assembly was conducted to recover as many transcripts as possible from the species of *Peromyscus* without a genome available. The genome-guided and de novo assemblies for each sample were combined using Transfuse v0.5.0 (https://github.com/cboursnell/transfuse; last accessed May 24, 2019.) for further analyses. The combined transcriptomes were analyzed for annotation completeness using BUSCO v3 (Simão et al. 2015; Waterhouse et al. 2018). The vertebrata_odb9 database was implemented in the BUSCO analysis and the lineage was set to “mouse.”

To discover unknown transcript splice sites, reads were aligned back to the genome using the coordinate sorted BAM approach in the program TopHat v2.1.2 (Trapnell et al. 2009). To extract the best open reading frames, the program Transdecoder v5.5.0 was used (Haas et al. 2013). BLAST (Camacho et al. 2009) was implemented for protein domain identification by comparing transcripts to the *M. musculus* database.

### DE, KEGG, GO, and Gene Networks

To visualize overall trends of expression patterns in all eight transcriptomes, a heatmap was constructed in RStudio (RStudio Team 2015) using R v3.6.0 (R Development Core Team 2008). A principle component analysis (PCA) and plot were generated to further visualize clustering patterns of the transcriptomes using the R packages, DESeq2 v1.24.0 (Love et al 2014) and ggplot2 v3.2.0 (Wickham 2016). Next, to conduct three DE analyses, the package DESeq2 v1.24.0 (Love et al. 2014) was implemented in RStudio. Read counts were normalized and the *P*-adjusted value for the Benjamin–Hochberg (BH) method was set to <0.01, a higher stringency than normal to allow for only 1% false discovery rate. First, comparisons were conducted on testis versus liver to ensure that the two tissue types had DE profiles. Second, to determine if reproductive genes with similar functions are expressed differentially in a repeated fashion across clades, DE profiles were generated for clade comparisons (A vs. B) for testis. Third, to determine if nonreproductive genes are expressed differentially at random (in terms of functionality), DE profiles were generated for clade comparisons (A vs. B) for liver. The package NOISeq v2.28.0 (Tarazona et al. 2011, 2015) was implemented to conduct within clade comparisons (*P*. *attwateri* vs. *P*. *boylii*; *P*. *leucopus* vs. *P*. *maniculatus*). This package was chosen because it allows for DE comparisons of samples with no replicates.

KEGG pathways of genes and gene clusters were determined using the package clusterProfiler v3.12.0 (Yu et al. 2012) and the genome wide annotation for *M. musculus* (org.Mm.eg.db v3.8.2; Carlson 2019). The enrichKEGG function was implemented using the BH *P*-adjusted method (*P* value cutoff = 0.01; *q* value cutoff = 0.05). BP GO terms were identified for transcripts that were significant in the DE analyses using the enrichGO function (in conjunction with the simplify function to reduce redundancy in the results) in the clusterprofiler package in R (padjust method = BH; *P* value cutoff = 0.01; *q* value cutoff = 0.01).

The database STRING v11.0 (Szklarczyk et al. 2015) was used to search for interactions between transcripts (identified to genes) in both the liver and testis DE data sets. For this analysis, genes for *M. musculus* were used to visualize gene interactions because *Peromyscus* is not an option on the database. Only transcripts that had significant (*P* adjusted ≤ 0.01) DE in the DESeq2 analyses were used to visualize network interactions.

## Results

### Transcriptome Assembly and General Expression Patterns

**Fig. 2.**
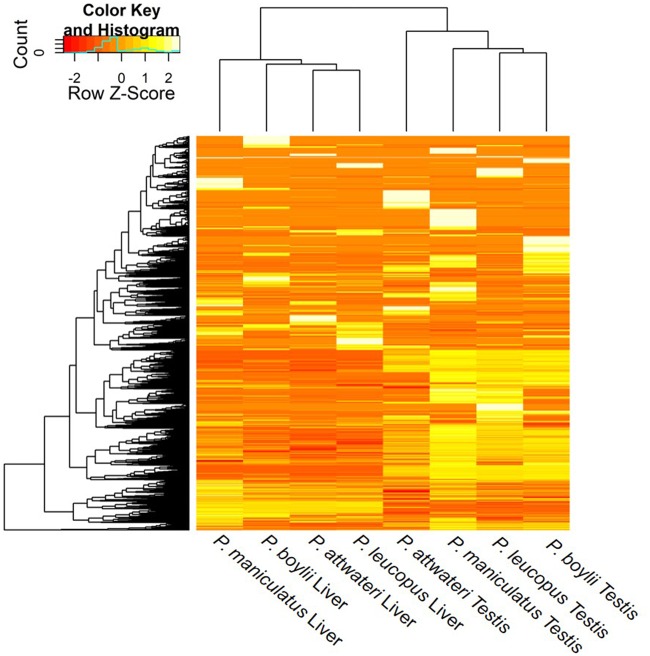
—Constructed heatmap of all liver and testes transcriptomes of *Peromyscus* generated using a genome-guided assembly. All liver transcriptomes exhibit similar transcript expression patterns, and all testes transcriptomes exhibit similar transcript expression patterns. The darker the color (red) the less expression, the lighter the color (white) the more expression of the gene in the transcriptome.

**Fig. 3.**
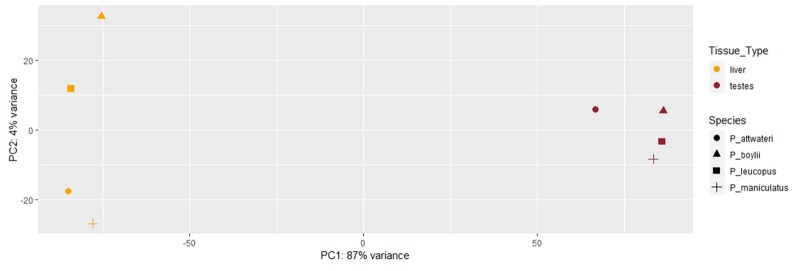
—A PCA plot depicting the similarities and dissimilarities of the liver and testis transcriptomes of four *Peromyscus* species.

The mean number of total reads assembled, across the two tissue types, was 19,311,295 (table 1). On average, there were higher numbers of assembled transcripts in the testis transcriptomes compared with the liver transcriptomes (table 1). The BUSCO results range from 41.3% to 76.9% completeness for transcriptome annotation (see table 2 for full BUSCO report). This range is within the reported range for transcriptomes from BUSCO (Simão et al. 2015; Waterhouse et al. 2018). Qualitatively, the heatmap (fig. 2) depicts that the testis and liver transcriptomes have more similar expression patterns within as compared with between tissue types. The PCA plot depicts that there are two clusters among the transcriptomes, placing liver samples in one cluster and testis samples in another cluster (fig. 3). This indicates that samples have more similar gene expression profiles within tissue types compared with between tissue types.

**Table 2:** The Complete Summary of Values Reported for the BUSCO Analysis

Species	Tissue Type	Complete	Single Copy	Duplicated	Fragmented	Missing
*Peromyscus attwateri*	Liver	41.3	27.1	14.2	25.9	32.8
	Testis	45.0	36.4	8.6	29.6	25.4
*Peromyscus boylii*	Liver	50.8	40.6	10.2	22.6	26.6
	Testis	67.8	43.4	24.4	15.2	17.0
*Peromyscus leucopus*	Liver	41.8	30.1	11.7	28.0	30.2
	Testis	64.7	36.6	28.1	17.4	17.9
*Peromyscus maniculatus*	Liver	54.8	29.5	25.3	20.5	24.7
	Testis	76.9	30.0	46.9	11.6	11.5

Note.—The total number of BUSCOs was 2,586. The values are reported as percentages.

### DE Analysis: Liver versus Testis

DE analyses were conducted to compare the expression profiles of the liver transcriptomes with the testis transcriptomes. The top ten most highly differentially expressed genes (based on log 2-fold change values) identified in the testis relative to the liver are thought to function in reproductive processes based on known GO terms associated with the genes and/or have documented overexpression or exclusive expression in testis (*Fhl1*, *Ace*, *Airp*, *Samd4a*, *Larp4*, *Ppm1d*, *Azin2*, *Prr30*, *Gykl1*, and *Axdnd1*). These reproductive roles include spermatogenesis, sperm motility, sperm development, and cell cycle processes. Although these results are expected, they suggest that the constructed transcriptomes were not cross-contaminated between the tissue types, and therefore can be used in further analyses.

### DE Analysis: A versus B

**Fig. 4.**
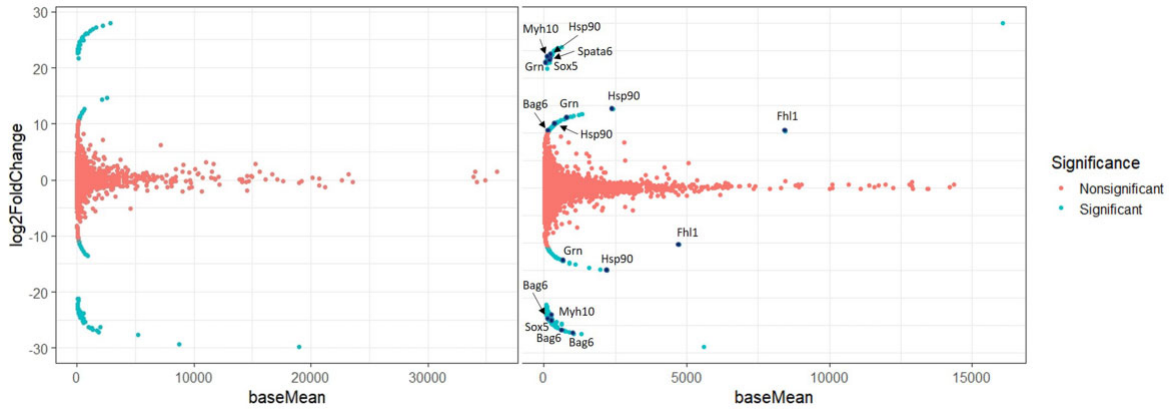
—DE of liver (left) and testis (right) transcriptomes of clade A versus clade B. baseMean, mean of normalized counts of all samples; log 2-fold change, log 2 scale to minimize differences between samples and represents change in upregulation or downregulation of DE of the transcript. Blue dots represent differentially expressed genes with a *P* adjusted ≤ 0.01 and pink dots represent differentially expressed genes with a *P* adjusted > 0.01. Genes mentioned in the Discussion section of this article are labeled in the DE plot of testis.

Results indicated that 252 transcripts had significant DE (*P* adjusted ≤ 0.01) in the liver data set (fig. 4*a*). There were 126 transcripts with higher expression in clade A and 126 transcripts with higher expression in clade B. Results indicated a larger number of transcripts (657) that were differentially expressed (*P* adjusted ≤ 0.01) in the testis data set (fig. 4*b*). There were 423 transcripts that exhibited significantly higher expression in the clade A compared with clade B, whereas 234 transcripts exhibited the reverse. To compare the ratios of DE transcripts in testis (657/14,600) versus liver (252/9,114), a two-sample test for equality of proportions with continuity correction (prop.test function) was conducted in R (*Χ*^2^ = 45.354, *P* value = 1.65e-11).

Transcripts were assigned UniProt identifications in the original BLAST to the *M. musculus* database. After the DE analysis, the UniProt IDs were converted to gene names. This revealed 45 genes that contained DE transcripts/isoforms in each group (A vs. B) in the testis analysis. For example, the gene *Bag6/Bat3* had three transcripts with unique UniProt IDs highly expressed in group A, whereas a fourth transcript with a unique UniProt ID was highly expressed in group B. There has been documentation of unique isoforms, products of alternative transcripts, for the *Bag6/Bat3* gene (Kämper et al. 2012; Binici and Koch 2014). Fragments, isoforms, and variants encoded by the same gene have separate entries and UniProt IDs. Therefore, all 45 genes with DE transcripts may not represent unique isoforms, however many of the genes were determined to represent alternative transcripts or isoforms (as identified through literature searches of individual gene names). Alternatively, there were 13 genes with DE transcripts/isoforms in the liver analysis.

### DE Analysis: Intraclade Comparisons

DE analyses were conducted for intraclade comparisons. The NOISeq command was used with no replicates, normalization set as reads per kilobase million. The percentage of reads used for each simulated sample was pnr = 0.2, and number of simulations was set as 10. The NOISeq analysis revealed that *P*. *attwateri* versus *P*. *boylii* contained 29.9% of DE genes in the liver data set simulations and 47.2% DE genes in the testis data set simulations. This suggests that for clade A, the two samples are more similar in the liver data set. Further, clade B (comparing *P*. *leucopus* to *P*. *maniculatus*) contained 34.9% DE genes in the liver data set simulations and 28.9% DE genes in the testis data set simulations. It should be noted that this statistical test should not be heavily relied on because no replicates are used in this comparison. Rather, the NOISeq analysis is implemented for quality control by creating a null or noise distribution of read counts within a sample (Tarazona et al. 2011).

### KEGG Annotation

The package clusterProfiler was used to determine KEGG pathways associated with DE transcripts (*P* adjusted ≤ 0.01). The top KEGG pathways represented in this analysis were peroxisome proliferator-activated receptors (PPAR) signaling pathway, chemical carcinogenesis, complement and coagulation cascades, peroxisome, and steroid hormone biosynthesis indicating that genes expressed in both liver and testes function in these pathways (supplementary file 1a, Supplementary Material online). However, there were 49 enriched KEGG annotations reported in this analysis (*P* value cutoff = 0.01; *q* value cutoff = 0.05), and 18.29% of UniProt IDs mapped to Entrez IDs and subsequent KEGG annotations.

KEGG pathways were determined for both the testis and liver comparisons of clades A and B. In the liver comparison, there were five enriched KEGG annotations (*P* value cutoff = 0.01; *q* value cutoff = 0.05) among the significant DE transcripts, and 50% of transcripts failed to map to KEGG annotations (supplementary file 1b, Supplementary Material online). The enriched KEGG annotations in liver were steroid hormone biosynthesis, complement and coagulation cascades, PPAR signaling pathway, chemical carcinogenesis, and insulin signaling pathway. There was one enriched KEGG annotation (RNA transport) in the testis DE analysis (*P* value cutoff = 0.01; *q* value cutoff = 0.05). However, only 50.68% of the UniProt IDs mapped to Entrez IDs and subsequent KEGG annotations (supplementary file 1c, Supplementary Material online).

### Gene Ontologies

There were 133 significant (*P* adjusted ≤ 0.01) BP GO terms enriched from the testis transcriptomes compared with the liver transcriptomes (supplementary file 2a, Supplementary Material online). It should be noted that many of the UniProt identifications assigned to transcripts do not have GO term identifications assigned in the *Mus* database, to date. Of the 86,153 UniProt identifications listed in the *M. musculus* UniProt database, 69,134 UniProt identifications are still unreviewed (UniProt Consortium 2019).

There were 185 significant (*P* adjusted ≤ 0.01) BP GO terms assigned to DE transcripts in the liver for the A versus B comparison using the program, clusterProfiler (supplementary file 2b, Supplementary Material online). The majority of BP GO terms associated with liver includes functions in metabolic and catabolic processes. Conversely, 128 significant (*P* adjusted ≤ 0.01) BP GO terms assigned to DE transcripts in the testis for the A versus B comparison (clusterProfiler), and most of these GO terms are associated with meiosis (cell cycle processes) or are associated with reproductive processes (sperm motility, acrosome assembly, and sperm–egg fusion) (supplementary file 2c, Supplementary Material online).

### Gene Networks

**Fig. 5.**
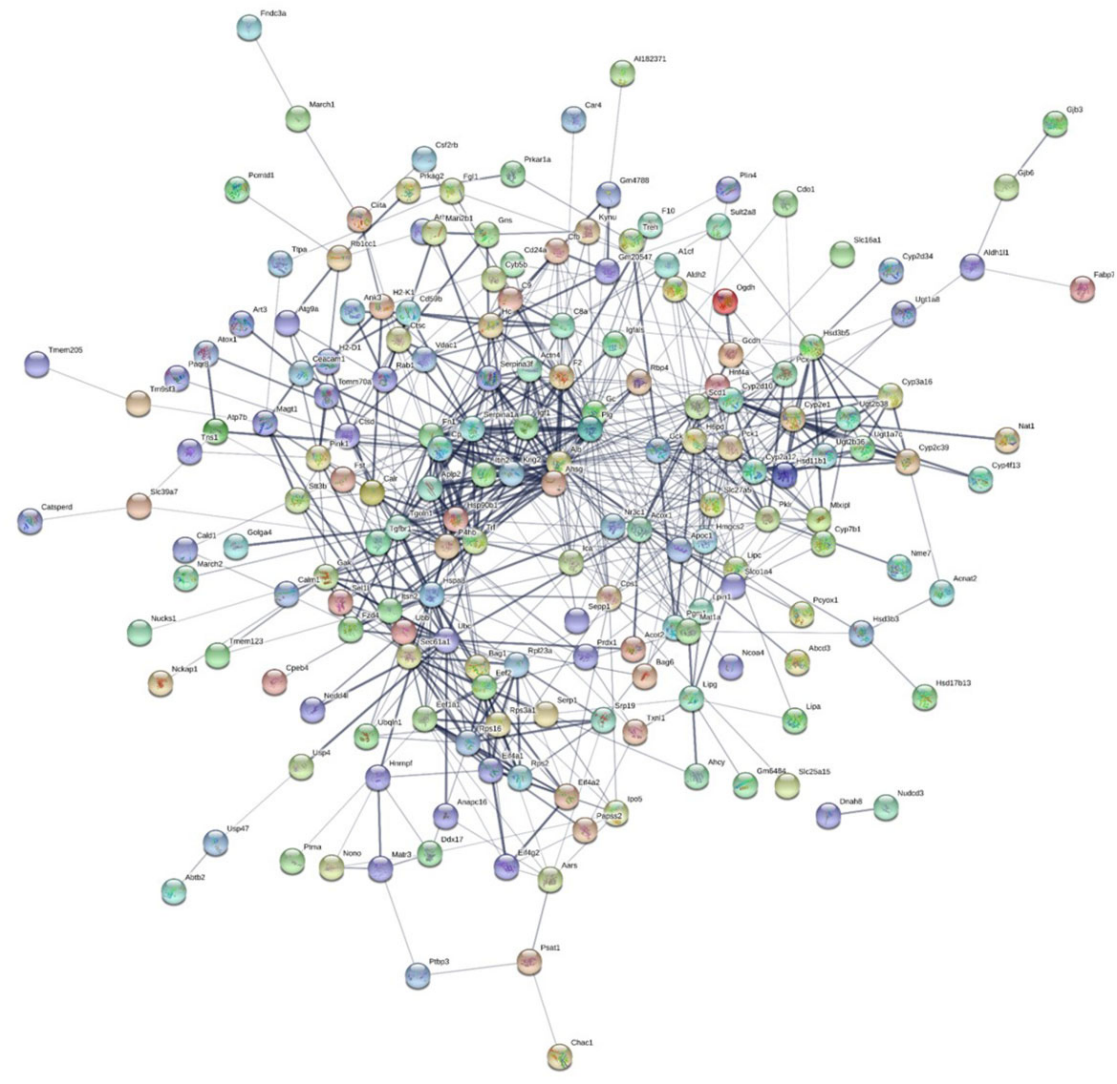
—Gene network analysis generated using the STRING database for DE genes in the liver transcriptomes. Genes that had no known interactions within the data set are not shown. There are three main clusters in DE genes of the liver.

**Fig. 6.**
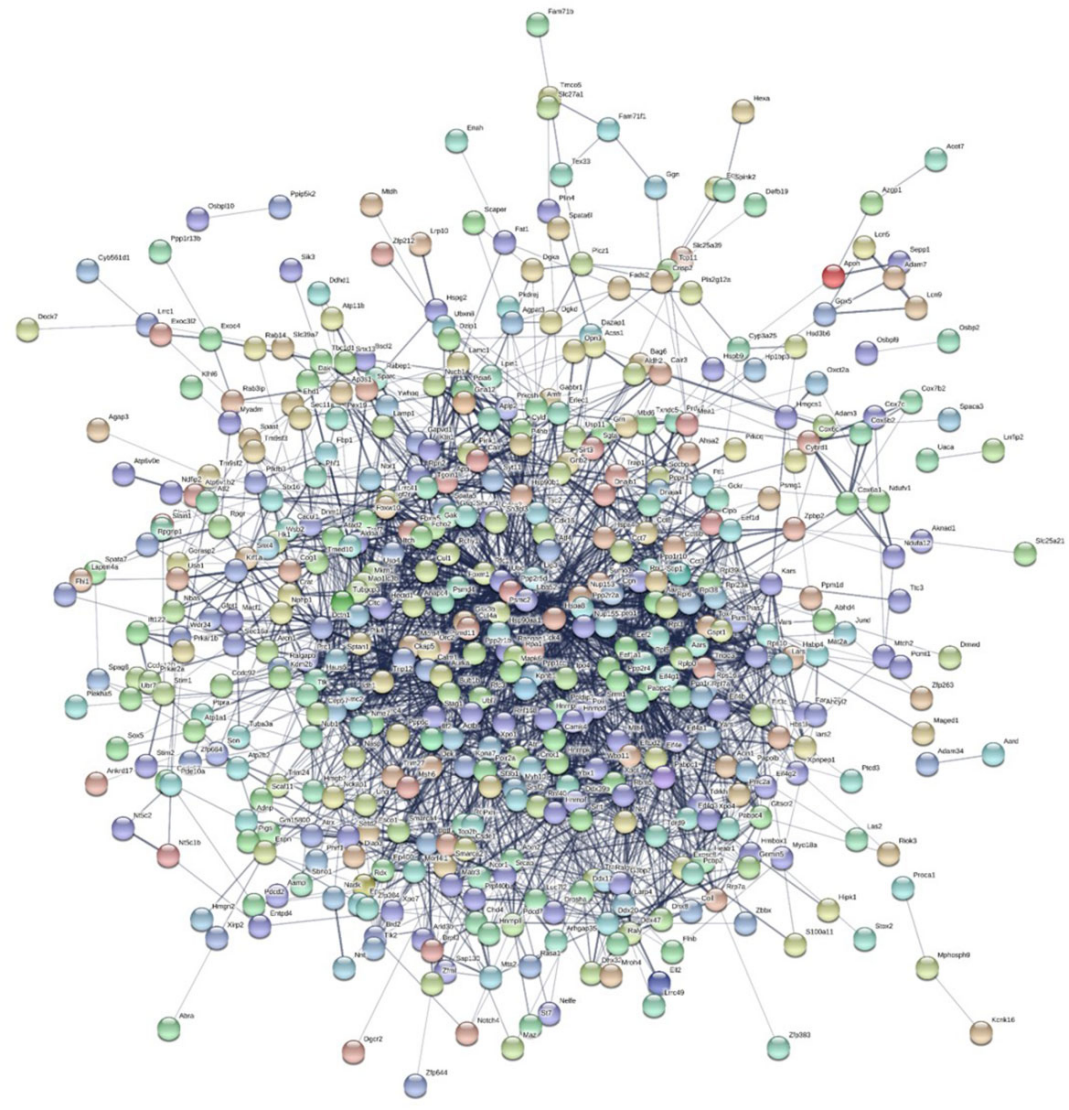
—Gene network analysis generated using the STRING database for DE genes in the testis transcriptomes. Genes that had no known interactions within the data set are not shown. There is one major cluster in DE genes of the testis and many of the genes had multiple interactions.

The STRING database was used to draw the interactions with all transcripts overexpressed in the liver data set (fig. 5). There were three main clusters of protein interactions among the liver data set with significantly more interactions than expected (protein-protein interaction [PPI] enrichment *P* value < 1.0e-16; expected number of edges = 324; actual number of edges = 708). The three main clusters included genes that functioned in 1) monocarboxylic acid metabolic processes, 2) cellular amide metabolic processes, and 3) response to stress. Interactions were also drawn for DE transcripts in the testis data set (fig. 6). The analysis had a higher number of interactions than expected in a random group of genes in a genome. The expected number of edges was 1,837; whereas, the actual number of edges for this data set was 2,732 (PPI enrichment *P* value < 1.0e-16). There was only one main cluster of gene interactions in comparison to the three clusters discovered in the liver data set. This suggests that the DE transcripts in the testis data set have more broad interactions with each other and possibly have more functional roles in common with each other.

## Discussion

Results of this study revealed that gene expression in testis of *Peromyscus* is more variable than in the liver. As expected, the top expressed genes from the testis transcriptomes in this analysis indicated that they perform reproductive functions. These results confirm one of the primary predictions of this study in that DE levels would be identified in liver versus testis transcriptomes.

There were fewer DE transcripts in the liver transcriptomes (*n* = 252). This suggests that overall interspecific gene expression in the liver is less variable. For the DE comparison in the testis data set, there were 657 transcripts that were differentially expressed (*P* adjusted ≤ 0.01). A chi-square test revealed that the number of DE transcripts in the testis compared with liver was significantly different (*P* < 0.05). This finding supports our first prediction that there were a greater number of DE genes in the testis data set than in the liver data set.

Further, there were more BP GO terms associated with DE transcripts in the liver data set compared with the testis data set. Most of these terms were associated with metabolic and catabolic processes. In the testis data set, most of the BP GO terms enriched were associated with reproductive processes. For instance, many GO terms were associated with meiosis and cell cycle processes (chromosome segregation, centrosome cycle, DNA damage checkpoint, among others). Meiosis is an important process of sperm maturation through spermatogenesis (Clermont 1972; Eddy 1998). Further, GO terms also were associated with motility, such as flagellum-dependent cell motility. Motility is important to propel sperm toward the egg, and studies have shown that sperm motility varies among species based on many factors such as metabolic capabilities (Turner 2006). According to our results, DE genes that play “nonreproductive” roles may be differentially expressed in a stochastic manner (as measured by functionality); whereas DE genes that are involved in reproductive processes were differentially expressed in a repeated fashion, as predicted. However, given the constraints of this study, we cannot determine for certain if DE genes in the liver were due to genetic drift or selective pressures. Intraspecies comparisons involving multiple replicates, as well as those involving multiple species, are needed to determine if DE of nonreproductive genes is expressed in a stochastic manner across the *Peromyscus* phylogeny.

The only KEGG annotation assigned to DE genes in the testis data set was RNA transport. Although this KEGG annotation is vague, it provides several avenues of discussion. For instance, transporting mRNA from the nucleus is imperative for gene expression. Further, ribonucleoproteins will transport inactive mRNA to be used in later stages of spermatogenesis (Kreysing et al. 1994). Examining more transcriptomes for multisample comparisons may result in an increased number of KEGG annotations associated with DE transcripts in testis.

The simplify command was used to reduce redundancy in the results of GO terms enriched in the testis data set. Upon examination of the BP GO terms, many of these terms are associated with proteolysis (e.g., protein modification by small protein removal). Proteolysis is an important process in the fusion of sperm and egg (Blobel et al. 1990; Phelps et al. 1990; Sutovsky 2003). Further, many GO terms were associated with the Golgi apparatus (Golgi vesicle transport, Golgi organization, and Golgi vesicle building). The Golgi apparatus is important in facilitating the movement of proteins associated with the acrosome assembly to the surface of the sperm head (Moreno et al. 2000). This is further evidence that DE transcripts have a role in the acrosome and sperm–egg fusion.

Several genes that were differentially expressed in the testis data set represented different isoforms of the same gene (45 genes in the 657 DE transcripts). Included in this list is Heat shock protein 90, *Hsp90*. There are several known isoforms of *Hsp90*. Two isoforms, in particular, *Hsp90aa1* and *Hsp90b1* are localized to the sperm head, suggesting a role in sperm–egg interactions (Maselli et al. 2014). DE of *Hsp90* isoforms could result in alternative zona pellucida (ZP) receptors in different species of *Peromyscus*. Another ZP-binding gene with DE isoforms, *Grn*, also known as acrogranin forms a complex with ADAM15 and has been shown to localize on the sperm surface playing a role in the sperm–egg binding process (Pastén et al. 2014).

Other genes that have differentially expressed isoforms play a role in mitosis and/or meiosis (*Cnot1*, *Tox4*, *Zfp37*, and *Usp4*). DE isoforms of spermatogenic genes also included *Bag6*, *Csde1*, *Fhl1*, *Mkrn1*, *Sec11a*, *Smarca2*, and *Tra2a*. Several other genes with DE isoforms play a role in acrosome biogenesis and/or sperm capacitation (*Gorasp2*, *Gopc*, *P4hb1*, and *Trap1*). Egg coats may vary among species of *Peromyscus* and may be impenetrable by other species’ sperm depending on the enzymes contained in the acrosomes of sperm.

Another gene of interest with DE isoforms is *Myh10*. This gene interacts with *Spata6* which is required for the sperm connecting piece (essential for linking the flagellum to the sperm head). Several genes with DE isoforms are involved in sperm motility, *Tra2a*, *Sox5*, and *Ralgds.* There has been evidence that genes involved in sperm motility are differentially expressed in different species. For instance, *Prkar1a*, a protein kinase, was shown to localize to the midpiece of *Peromyscus* sperm (associated with sperm motility) and was differentially expressed among two species, one with a short midpiece and one with a long midpiece (Fisher et al. 2016). Similarly, *Spag6* has been shown to localize to the sperm tail (including the midpiece) and interacts with *Sox5* (Kiselak et al. 2010), suggesting that DE in *Sox5* may lead to midpiece variations among the species of *Peromyscus*.

**Fig. 7.**
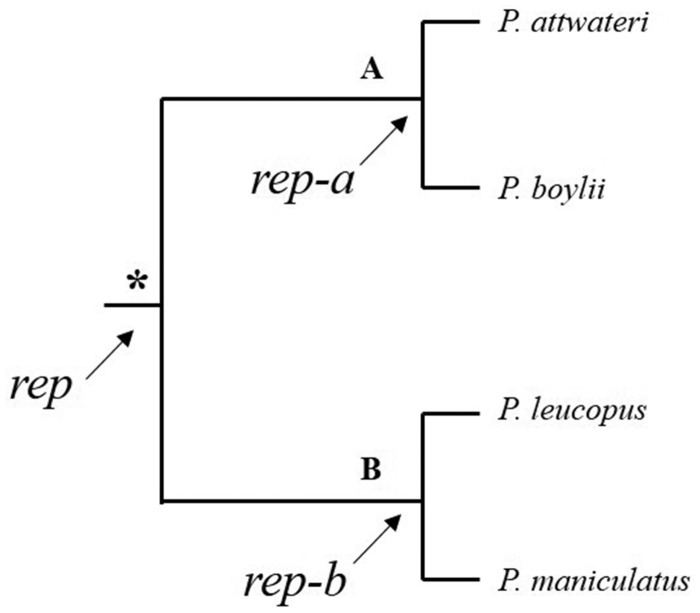
—Simplified phylogenetic tree showing common ancestors of all four taxa analyzed in this study. **A** represents common ancestor of *P*. *attwateri* and *P*. *boylii* and **B** represents common ancestor of *P*. *leucopus* and *P*. *maniculatus*. The asterisk (*) denotes the common ancestor for **A** and **B**. *rep*, “reproductive” gene; *a* and *b*, isoforms of *rep*. We hypothesize, based on results, that alternative transcripts of reproductive genes were differentially expressed in the common ancestor of **A** and **B** that eventually led to the speciation of **A** and **B** and subsequent speciation events of extant species of *Peromyscus*.

Alternative splicing can potentially play a role in adaptation and speciation of closely related species (Ast 2004; Xing and Lee 2006; Harr and Turner 2010). For example, Harr et al. (2006) were able to determine that alternative transcripts of a gene, *Mkk7*, in testis of *M. musculus domesticus* and *M. musculus musculus* were differentially expressed between the two subspecies and may be a plausible target for adaptive differences. Perhaps, the ancestral species of *Peromyscus* started to express alternative transcripts of ZP-binding, spermatogenic, acrosomal, and/or sperm motility genes, leading to the speciation events that occurred in the genus (fig. 7).

Many of the UniProt identifications assigned to transcripts did not have GO terms assigned in the *Mus* database. It is possible that these genes have not been classified in the *Mus* genome. To date, the *Mus* genome has over 17,000 reviewed and annotated UniProt entries (UniProt Consortium 2019). Many of the *Peromyscus* transcripts obtained herein were not assigned gene names when compared with the *Mus* genome. *Mus* is a murid rodent (family Muridae) whereas *Peromyscus* is in the Cricetidae family and they have been separated for ∼22–25 Myr (Steppan et al. 2004; Fabre et al. 2012). The transcripts in the *Peromyscus* transcriptomes of this project may differ from known *Mus* genes enough to not meet the 65% threshold set in the blast parameters described in the Materials and Methods section. This could impact our results by decreasing the number of transcripts that BLAST to known identifications but we do not think this is the case because we still have a substantial amount of transcripts that mapped to known identifications, and there are a small number of orphan transcripts. These orphan transcripts may represent de novo transcripts that are unique to *Peromyscus*.

Although we cannot conclude for certain what caused the numerous speciation events of *Peromyscus*, we can hypothesize that in an ancestor to nodes A and B, a group of reproductive genes began to express alternative transcripts or exhibit variable expression patterns that led to the speciation of nodes A and B (fig. 7). This may reveal potential mode(s) of reproductive isolation in the long-term evolutionary trajectories of *Peromyscus*. Speciation processes are not simple and speciation events of *Peromyscus* most likely were due to multiple factors over millions of years. The cross-section of genes identified herein is just the beginning of efforts to discover genes that played a role in the speciation processes in *Peromyscus*. Although we do not have all the answers to what caused the numerous speciation events, we can start to elucidate on the possible reproductive isolating mechanisms in this speciose group.

Future studies are needed to corroborate the findings reported herein. This may include examining the testis transcriptomes of more species of *Peromyscus*, including sister species and species with longer divergence times. This would provide more data and the opportunity to analyze DE within and between sister species. If a suite of genes were identified as being involved in major speciation events (specifically, taxa comprising a clade of organisms that resulted from a rapid, adaptive radiation), then we would expect that those events are repeated throughout the evolutionary history of that clade. Examining genes of interest through quantitative polymerase chain reaction or other molecular techniques would provide further evidence of findings reported in this article. Further, more examination is needed on nonreproductive genes to determine if such genes evolved and are expressed in a stochastic manner, as predicted, or if they are also expressed in a repeated fashion across the genus. If nonreproductive genes that have similar functions are expressed differentially in a repeated fashion across clades, then speciation of *Peromyscus* occurred from more than just reproductive isolation events. Other studies could involve the examination of the divergence of gene sequences that are potentially involved in the reproductive isolations of *Peromyscus* to determine if genes that presumably play reproductive roles are positively selected for across the *Peromyscus* phylogeny.

## Supplementary Material

Supplementary data are available at *Genome Biology and Evolution* online.

## Supplementary Material

evz280_Supplementary_Data

## Notes

**Data deposition:** This project has been deposited at NCBI SRA under the accession PRJNA522999.
